# Triglyceride-glucose index and combined indicators: effective indicators for screening NAFLD in snoring patients

**DOI:** 10.1186/s12890-024-03166-8

**Published:** 2024-07-24

**Authors:** Yuqing Cai, Jia Chen, Xiaoyu Deng, Biying Wang, Jiefeng Huang, Ningfang Lian

**Affiliations:** 1 Department of Respiratory and Critical Care Medicine, Respiratory Disease Research Institute, The First Affiliated Hospital, Fujian Medical University, Fuzhou, 350005 China; 2 Department of Respiratory and Critical Care Medicine, National Regional Medical Center, Binhai Campus of the First Affiliated Hospital, Fujian Medical University, Fuzhou, 350212 China

**Keywords:** Triglyceride-glucose index, Snoring, NAFLD, Risk factor

## Abstract

**Aims:**

Nonalcoholic fatty liver disease (NAFLD) is a common complication in snoring patients, especially in patients with obstructive sleep apnea syndrome (OSA). Triglyceride-glucose (TyG) index was a simple indicator of metabolic status and a surrogate marker of insulin resistance. This study aimed to explore the relationship between NAFLD and TyG index in snoring patients.

**Methods:**

A retrospective study was conducted. The successive snoring patients enrolled in the Sleep Center of the First Affiliated Hospital of Fujian Medical University and had abdominal ultrasonography were included. The clinical characteristics of patients in different quartile TyG groups were compared. The relationship of the TyG index and NAFLD were valued via logistic regression models and restricted cubic spline analysis. The value of TyG index in predicting NAFLD was determined by receiver operating characteristic curve (ROC curve).

**Results:**

A total of 463 NAFLD cases were found among the 654 snoring patients. TyG index was a risk factor of NAFLD in snoring patients (OR = 2.38, 95% CI = 1.71–3.36). The risk of NAFLD was much higher in patients with the highest quartile of TyG index (OR = 5.12, 95% CI = 2.85–9.22), compared with the lowest quartile group. Restricted cubic spline (RCS) analysis showed a significant dose-response relationship between TyG index and risk of NAFLD (*p* for non-linearity < 0.001). A combination of TyG, neck circumference and ESS score presented the acceptable AUC for the detection of NAFLD in snoring patients (0.746, 95% CI 0.701–0.790, *p* < 0.001).

**Conclusion:**

The TyG index was a risk factor of NAFLD in snoring patients. A combination of TyG, neck circumferences and ESS score could act as a convenient and effective indicator for screening NAFLD in snoring patients.

## Introduction

Nonalcoholic fatty liver disease (NAFLD), as one of the most common chronic liver diseases, can affect approximately 25% of the global population [1, 2]. Currently, NAFLD is considered as the liver manifestation of metabolic syndrome, closely related to various metabolic diseases such as obesity, dyslipidaemia and type 2 diabetes mellitus [3, 4]. With the changes of life style, the prevalence of NAFLD is generally increasing, which undoubtedly increases the global economic burden [5, 6].

Epidemiological evidences suggest that snoring, as a common clinical manifestation, also plays an important role in various metabolic related diseases [7, 8]. Among them, the snorers have a higher probability of dyslipidemia [9]. And a study from China showed that snoring may be related to the prevalence and the 10-year incidence of NAFLD [10]. Meanwhile, snoring is the common clinical presenting symptoms of obstructive sleep apnea syndrome (OSA) [11]. OSA and NAFLD promote and interact with each other [12]. Therefore, it is necessary to pay more attention on NAFLD risk screening in snoring patients.

Triglyceride-glucose (TyG) index is closely related to development and progression of liver steatosis and liver fibrosis [13]. A growing body of research indicates that TyG index could be an effective noninvasive method for the identification of NAFLD [14–16]. While, most of the above studies are based on subjects undergoing physical examinations, lacking more specific research on snoring subjects. As mentioned above, the snorers have a higher prevalence and incidence of NAFLD [10, 17]. Thus, the purpose of the present study is to further explore the relationship between NAFLD and TyG index in the snorers.

## Methods

### Study populations

A retrospective cross-sectional study was conducted. This study complied with the Declaration of Helsinki. The study design was approved by the Ethics Committee of the First Affiliated Hospital of Fujian Medical University. All consecutive snoring patients admitted to the Sleep Center, First Affiliated Hospital of Fujian Medical University were enrolled. The enrollment period of the study was between January 1, 2016 and December 31, 2019.

The including criteria were as follows: (1) Snoring patients suspected to be with sleep apnea. (2) Aged 18–80 years. (3) With the results of abdominal ultrasonography during hospitalization. (4) with the results of completed overnight polysomnography. (5) With completed clinical data. Subjects with various viral hepatitis, liver malignancy, alcoholic liver disease, or other known chronic liver disease were excluded.

The anthropometric parameters and Epworth Sleepiness Scale (ESS) were obtained from the electronic medical record system.

Sleep-related data were obtained from polysomnography monitoring reports finished via polysomnography respiratory monitoring system (Condi Australia). The data included AHI, lowest oxygen saturation (LSaO2), mean oxygen saturation (MSaO2), the percentage of sleep time with SpO2 < 90% (T90%) and oxygen desaturation index (ODI). The diagnosis of apnea, hypopnea and OSA were based on 2012 American Academy of Sleep Medicine (AASM) criteria [18]. As follows: AHI < 5 events/h, without OSA; AHI 5–14.9 events/h, mild OSA; AHI 15.0–29.9 events/h, moderate OSA; AHI ≥ 30 events/h; severe OSA.

Triglycerides, blood glucose, liver function, blood routine test were performed in fasting conditions on the next morning after Polysomnography. The TyG index was calculated as Ln [TG (mg/dL) × FBG (mg/dL)/2].

The diagnosis of NAFLD in the study was diagnosed via the results of abdominal ultrasonography after excluding the subjects with excessive alcohol consumption [3].

### Statistical analysis

Baseline characteristics of all snoring patients were described across TyG index variability quartiles. Mean ± SD or median (interquartile range) were used to describe continuous variables. Count (proportion) was used to describe categorical variables. The Kruskal–Wallis test or one-way ANOVA were used to compare the continuous variables. χ2 test were used to compare categorical variables. The relationship of the TyG index and NAFLD were valued via logistic regression models and restricted cubic spline analysis. The value of TyG index and combined prediction index in predicting NAFLD in snoring patients was determined by receiver operating characteristic curve (ROC curve). A *p* < 0.05 was defined as a statistically significant difference. Statistical analysis and plotting were conducted by R 3.6.2 (https://www.r-project.org/).

## Results

### Baseline characteristics of snoring patients

Anthropometric parameters and sleep related parameters were summarized in Table 1. A total of 654 snoring patients were included, of whom 522 (80%) were male, 581 (89%) suffered from OSA, with the average age of (51 ± 14) years, average AHI of (33 ± 25) events/h. The mean value of TyG index was (8.87 ± 0.67), and the ranges of TyG index for quartiles 1–4 were Quartile1 (7.32–8.43), Quartile 2 (8.44–8.81), Quartile 3 (8.82–9.27) and Quartile 4 (9.28–11.29), respectively. Patients in the highest quartile of TyG index were slightly younger, had higher prevalence of diabetes, more frequently smokers, and had higher levels of BMI, neck circumferences, waistlines and diastolic blood pressure. Patients in the highest quartile of TyG index also had higher levels of AHI, ESS, TS90%, MSaO2(%) and ODI.

**Table 1:** The basic characteristics and sleep parameters among different TyG index groups

	Overall (7.32–11.29)	Quartile1 (7.32–8.43)	Quartile2 (8.44–8.81)	Quartile3 (8.82–9.27)	Quartile4 (9.28–11.29)	*p* value
Male(%)	522 (80%)	126 (76%)	130 (80%)	131 (81%)	132 (83%)	0.500
Age(years)	51 (14)	53 (15)	52 (16)	51 (14)	48 (13)	0.001
Hypertension (%)	335 (51%)	79 (48%)	83 (51%)	89 (55%)	83 (52%)	0.600
Diabetes (%)	114 (17%)	15 (9.1%)	27 (17%)	25 (16%)	46 (29%)	< 0.001
Smoke (%)	299 (30%)	42 (25%)	44 (26%)	48 (30%)	63 (40%)	0.026
BMI (kg/m2)	27.9 (10.0)	27.7 (18.0)	27.1 (5.2)	28.0 (3.5)	28.7 (5.7)	< 0.001
Neck Circumference (cm)	39.4 (3.7)	38.1 (3.7)	39.1 (3.3)	39.8 (3.3)	40.7 (4.1)	< 0.001
Waistlines (cm)	99 (12)	95 (13)	98 (11)	101 (11)	102 (11)	< 0.001
ESS	7.7 (5.2)	6.9 (4.7)	7.1 (5.4)	8.2 (5.1)	8.6 (5.6)	0.021
AHI	33 (25)	27 (21)	31 (24)	38 (26)	37 (27)	< 0.001
TS90%	45 (81)	28 (53)	40 (75)	53 (82)	61 (103)	0.003
LSaO2(%)	74 (14)	76 (13)	74 (14)	74 (14)	73 (14)	0.200
MSaO2(%)	92.4 (6.4)	92.7 (9.1)	93.0 (3.9)	92.1 (4.7)	91.7 (6.8)	0.004
ODI	27 (24)	21 (19)	25 (23)	31 (26)	32 (28)	0.001
SBP (mmHg)	134 (17)	132 (16)	131 (17)	136 (16)	135 (16)	0.056
DBP (mmHg)	82 (12)	81 (12)	80 (12)	83 (13)	85 (11)	< 0.001

Abbreviation: BMI, body mass index; ESS, Epworth Sleepiness Scale; AHI, apnea‑hypopnea index; TS90%, the percentage of sleep time with SpO2 < 90%; LSaO2, lowest oxygen saturation; MSaO2, mean oxygen saturation; ODI, oxygen desaturation index; SBP, systolic blood pressure; DBP, diastolic blood pressure; TyG index, Triglyceride-glucose index

The prevalence of NAFLD in the whole queue was 71%. The prevalences of NAFLD in quartiles 1–4 were 55%, 71%, 71% and 87%, respectively (*p* < 0.001). Besides metabolism-related indicators as shown in Table 2, the patients in higher TyG quartile trended to had higher levels of ALT, AST, ALP and GGT (all *p* < 0.05).

**Table 2:** Metabolic markers and liver function among different TyG index groups

	Overall	Quartile1	Quartile2	Quartile3	Quartile4	*p*-value
Creatinine (µmol/L)	71 (24)	70 (20)	70 (18)	73 (20)	72 (34)	0.057
Uric acid (µmol/L)	406 (192)	374 (92)	412 (335)	410 (106)	429 (124)	< 0.001
Glucose (mmol/l)	5.49 (1.78)	4.63 (0.61)	5.19 (0.91)	5.37 (1.18)	6.82 (2.78)	< 0.001
Cholesterol (mmol/l)	4.64 (1.00)	4.31 (1.03)	4.52 (0.89)	4.72 (0.93)	5.03 (1.03)	< 0.001
Triglyceride (mmol/l)	2.05 (1.69)	0.93 (0.24)	1.40 (0.25)	2.02 (0.42)	3.89 (2.51)	< 0.001
HDL (mmol/l)	1.04 (0.27)	1.19 (0.33)	1.04 (0.25)	1.00 (0.22)	0.94 (0.22)	< 0.001
LDL (mmol/l)	3.02 (0.98)	2.87 (0.97)	3.10 (0.87)	3.11 (0.84)	3.00 (1.20)	0.021
ALT (u/l)	37 (43)	33 (46)	34 (52)	36 (29)	45 (41)	< 0.001
AST (u/l)	25 (18)	26 (27)	24 (11)	25 (16)	27 (14)	0.035
ALP (u/l)	71 (26)	68 (19)	72 (36)	69 (25)	75 (22)	0.002
GGT (u/l)	47 (62)	31 (21)	42 (48)	47 (50)	68 (98)	< 0.001
White blood cell (× 10⁹ cells per L)	6.90 (2.46)	6.46 (2.05)	6.80 (1.92)	7.13 (3.44)	7.28 (2.07)	0.001
Neutrophil (× 10⁹ cells per L)	4.02 (1.67)	3.82 (1.51)	4.00 (1.56)	4.02 (1.80)	4.29 (1.78)	0.073
NAFLD (%)	463 (71%)	90 (55%)	116 (71%)	115 (71%)	139 (87%)	< 0.001
TyG index	8.87 (0.67)	8.11 (0.26)	8.63 (0.10)	9.03 (0.13)	9.76 (0.48)	< 0.001

Abbreviation: HDL, high density lipoproteincholesterol; LDL, low density lipoproteincholesterol; ALT, alanine aminotransferase; AST, aspartate aminotransferase; ALP, alkaline Phosphatase; GGT, gamma glutamyl transferase; NAFLD, Nonalcoholic fatty liver disease; TyG index, Triglyceride-glucose index

### The association between of TyG index and NAFLD

In snoring patients, a positive association between TyG index and NAFLD was observed. After fully adjusted by sex, age, smoke status, BMI, diabetes, HDL, cholesterol and oxygen desaturation index (Table 3), TyG index was still positively associated with the risk of NAFLD (odds ratio = 2.38, 95% CI: 1.71–3.36; *p* < 0.001). The continuous variables of TyG index were converted to a categorical variable (quartiles) to conduct sensitivity analysis. Compared with Quartile 1, the lowest TyG index quartile, the NAFLD risk increased with the levels of TyG index quartile. Patients in the highest quartile of TyG index showed an obviously increased risk of NAFLD (odds ratio = 5.12, 95% CI = 2.85–9.22) compared to those in the lowest quartile (*p* < 0.001). As shown in Table 3.

**Table 3:** The relationship between triglyceride-glucose index and non-alcoholic fatty liver disease in snoring patients

	Non-adjusted				Adjust I				Adjust II			
	OR	95% CI	*p* value		OR	(95% CI)	*p* value		OR	(95% CI)	*p* value	
**TyG index**	2.49	1.86, 3.41	< 0.001		2.45	1.82, 3.35	< 0.001		2.38	1.71, 3.36	< 0.001	
**TyG index quartile**												
Q1	Reference	—			Reference	—			Reference	—		
Q2	2.06	1.31, 3.26	0.002		2.04	1.29, 3.23	0.002		2.11	1.32, 3.39	0.002	
Q3	2.04	1.29, 3.24	0.002		2.01	1.28, 3.19	0.003		2.05	1.27, 3.36	0.004	
Q4	5.79	3.36, 10.40	< 0.001		5.61	3.25, 10.1	< 0.001		5.12	2.85, 9.55	< 0.001	
P for trend			< 0.001				< 0.001				< 0.001	

Data are presented as odds ratios, 95% confidence intervals, and *p* value

Non-adjusted model adjusts for: none

Adjust I model adjust for: Sex, Age (ys);

Adjust II model adjust for: Sex, Age (ys), smoke status, BMI, diabetes; HDL cholesterol (mg/dL) and oxygen desaturation index(ODI).

**Fig. 1:**
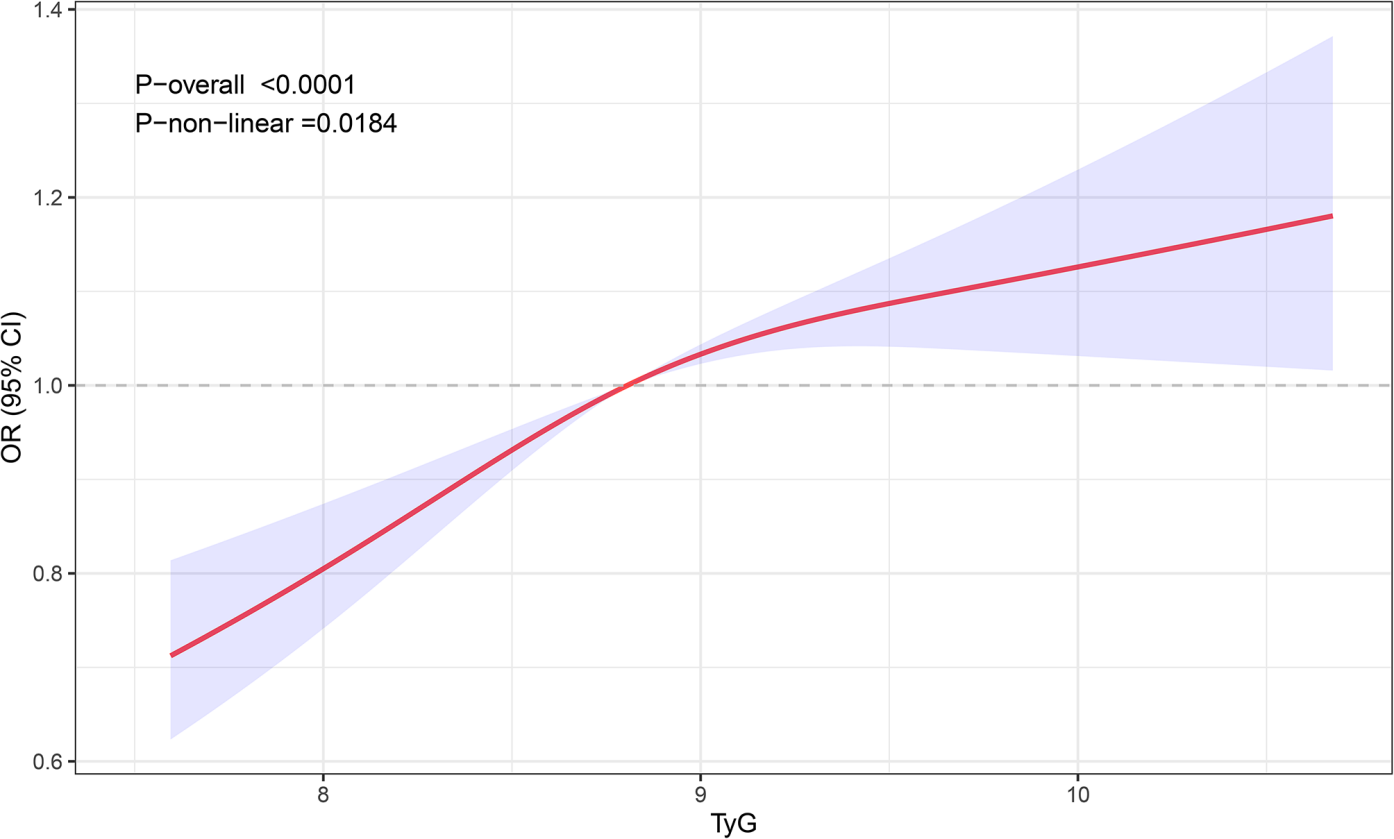
Restricted cubic spline fitting for the association between TyG index with non-alcoholic fatty liver disease in snoring patients

**Fig. 2:**
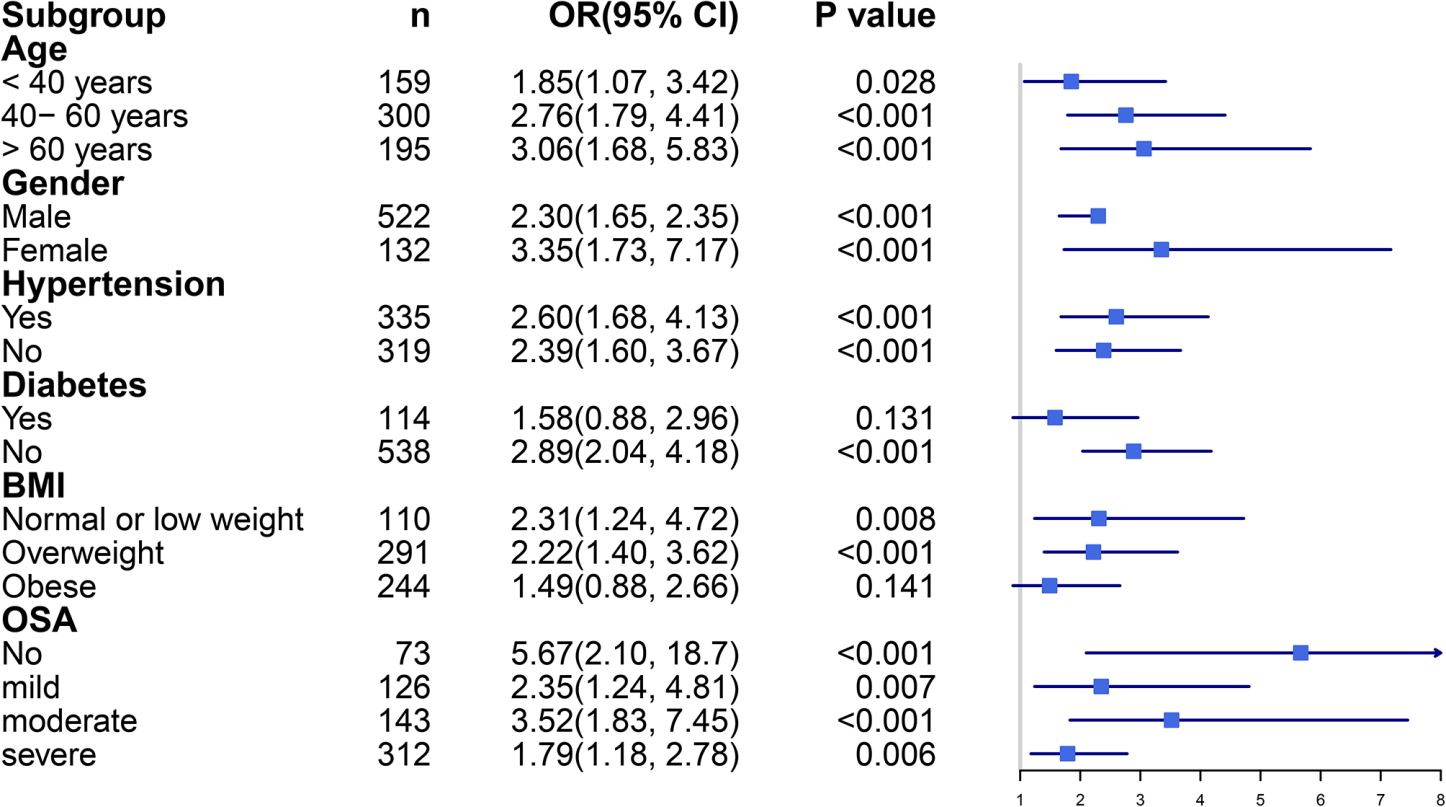
Subgroup analysis for the association between TyG index with non-alcoholic fatty liver disease in snoring patients

In addition, restricted cubic spline analysis (Fig. 1) showed the dose–response relationship between TyG index and the risk of NAFLD (*p* = 0.0184). We further performed subgroup analyses to confirm the robust association between TyG index and NAFLD in Fig. 2. The positive association of TyG index and NAFLD was in the most of the sub-populations. No significantly positive associations were found in patient with diabetes or obese patients.

### The value of TyG index and combined prediction index in predicting NAFLD in snoring patients

**Fig. 3:**
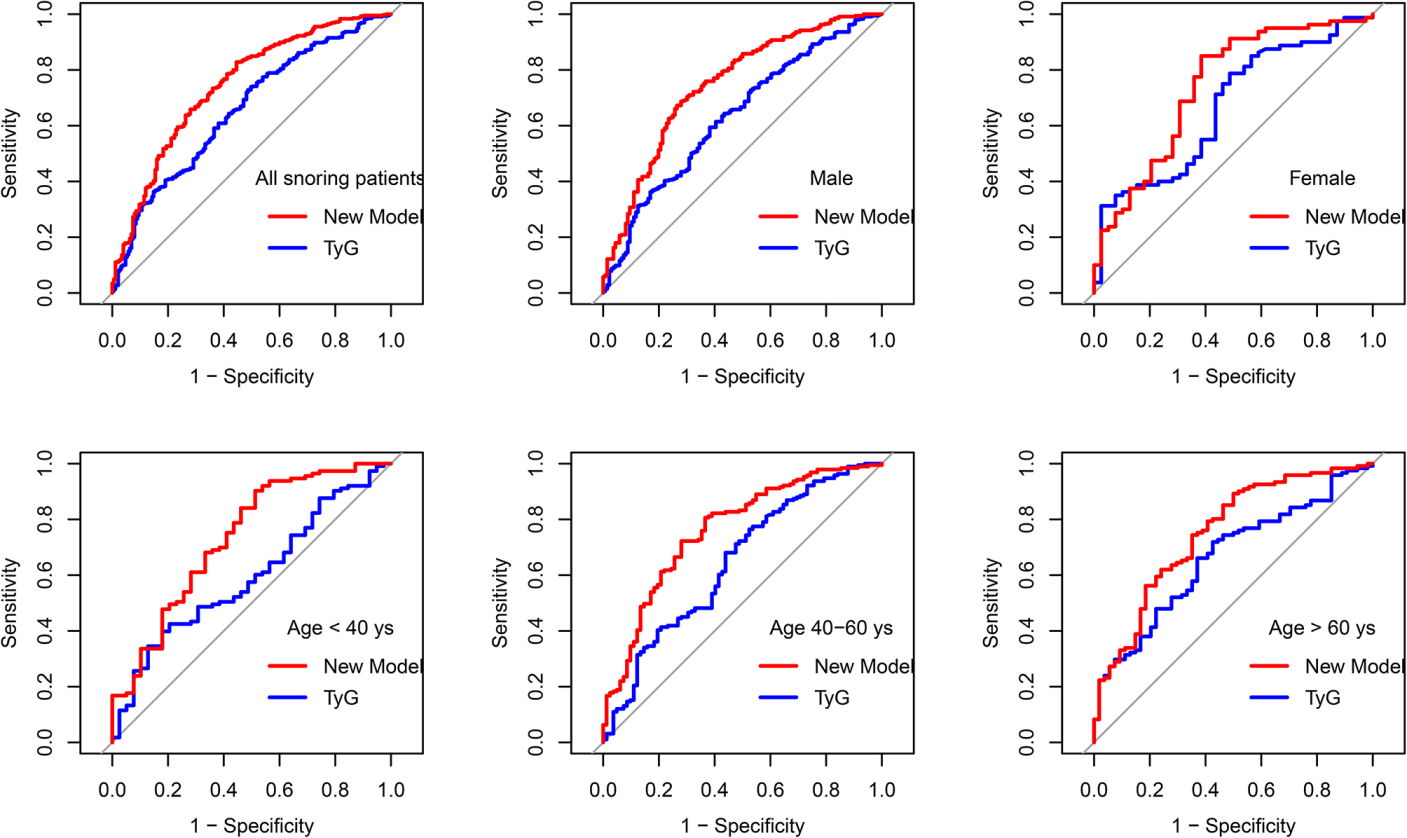
The value of TyG index and combined prediction index in predicting NAFLD in snoring patients. The combined prediction index of new mode was the combination of TyG, neck circumference and ESS. In all snoring patients, the AUCs for the TyG index and the combined prediction index were 0.643 (95% CI: 0.595–0.691, *p* < 0.001) and 0.746 (95% CI 0.701–0.701, *p* < 0.001) respectively. In Male, the AUCs for the TyG index and the combined prediction index were 0.635 (95% CI: 0.580–0.690, *p* < 0.001) and 0.749 (95% CI 0.699–0.798, *p* < 0.001) respectively. In Female, the AUCs for the TyG index and the combined prediction index were 0.667 (95% CI 0.565–0.770, *p* = 0.003) and 0.749 (95% CI 0.638–0.836, *p* < 0.001) respectively. In patients younger than 40 years, the AUCs for the TyG index and the combined prediction index were 0.611 (95% CI: 0.514–0.709, *p* = 0.037) and 0.728 (95% CI 0.631–0.625, *p* < 0.001) respectively. In patients aged 40–60 years, the AUCs for the TyG index and the combined prediction index were 0.632 (95% CI: 0.560–0.705, *p* < 0.001) and 0.750 (95% CI 0.686–0.813, *p* < 0.001) respectively. In patients older than 60 years, the AUCs for the TyG index and the combined prediction index were 0.675 (95% CI: 0.590–0.760, *p* < 0.001) and 0.741 (95% CI 0.659–0.823, *p* < 0.001) respectively

The value of TyG index in predicting NAFLD in snoring patients was investigated via ROC curve. In all snoring patients, the AUCs for the TyG index was 0.643 (95% CI: 0.595–0.691); while the combined prediction index of TyG index, neck circumference and ESS increased the AUC value to 0.746 (95% CI 0.701–0.701). To determine the stability of TyG index and combined indicators in predicting NAFLD in snoring population, ROC curves were plotted in different age subgroups and gender subgroups. As shown in Fig. 3, in different gender subgroups and subgroups of less than 40 years old, 40–60 years old and more than 60 years old, TyG and the combined detection index had similar predictive efficacy, and the AUC value fluctuated between 0.611 and 0.675 and 0.728–0.750, respectively (all *p* < 0.01).

## Discussion

In this study, the prevalence of NAFLD was higher in snorers with a higher level of TyG index. The higher TyG index was associated with a higher odds risk of NAFLD. In addition, a combination of TyG, neck circumference and ESS score presented the acceptable AUC for the detection of NAFLD in snoring patients.

Previous studies showed that sleep disorder, as a public health disease, may be closely related to insulin resistance [19].The TyG index is widely recognized as a reliable surrogate indicator for insulin resistance [20]. A study from NHANES 2005–2008 found that there was a linear dose-response relationship between TyG index and various sleep disorder, which is often accompanied by abnormal sleep related parameters [21]. In addition, multiple articles have confirmed the value of the TyG index in OSA [22–24]. This is consistent with our conclusion. In this study, different quartiles of TyG index were correlated with the severity of sleep related parameters, including AHI, ESS, TS90%, and so on. Meanwhile, the prevalence of NAFLD in the whole queue was 71%, and the prevalence of NAFLD was higher in snorers with a higher level of TyG index. NAFLD is common in the general population and affects up to 75% of obese individuals worldwide [25, 26]. And snoring is more common in obese individuals. The relationship between sleep and NAFLD is bidirectional, which indicate that more severe oxidative stress, systemic and vascular inflammation with endothelial dysfunction, and ischemia-reperfusion injury may contribute to the occurrence of NAFLD [27].

As we know, NAFLD encompasses a series of hepatic pathologies from simple hepatic steatosis to nonalcoholic steatohepatitis and cirrhosis, accompanied by the increase of ALT, AST and other hepatic enzymes [3]. Moreover, elevated serum levels of ALT and GGT were markers of oxidative stress and inflammation [28]. The level of ALT, AST, ALP and GGT trended to be higher in patients with higher TyG quartile in this study, which may indicate more severe oxidative stress and inflammation. The level of TyG index may act as a predictive indicator for liver disease progression, which is consistent with the conclusion of Zhang et al [15].

The Insulin resistance (IR) induces lipid accumulation in hepatocytes and leads to the occurrence of fatty liver [29]. TyG index takes into account insulin resistance and dyslipidemia, which are crucial metabolic mechanisms of NAFLD [12]. The relationship between TyG index and NAFLD in snorers was further confirmed in this study. In addition, it is worth mentioning that compared with the lowest quartile group, the risk of NAFLD in the highest quartile of TyG index increased by 5.12 times (OR = 5.12, 95% CI = 2.85–9.22). Early intervention measures such as lifestyle adjustment and medication treatment may have higher benefits for such patients. However, there is no significant positive correlation in subgroup analysis of diabetes or obese patients, which is inconsistent with some previous studies [30, 31]. Diabetes or obesity may affect the efficacy of TyG index in identifying snorers with high NAFLD risk. The different metabolic states and fat distribution of the included population may be potential mechanisms [32, 33]. Other reasons may be that diabetes and obesity take more interventions due to complications.

Currently, anthropometric parameters have been widely used as a simple and feasible tool for screening metabolic disorders in the general population. Compared to traditional indicators, indicators that reflect the fat distribution play a more important role in metabolic diseases [34]. Among them, the surrogate marker of central obesity, such as waist circumference and waist-to-height ratio, are closely related to visceral adipose tissue (VAT) [35]. However, studies have shown that the relationship between upper body subcutaneous adipose tissue and metabolic disorders is stronger than VAT [36]. And neck circumference (NC), a surrogate of upper body subcutaneous fat, had higher predictive value and was more feasible in assessing the risk of NAFLD than other anthropometric indicators mentioned above [34]. In addition, daytime sleepiness is common in patients with NAFLD and may be a contributing factor to the reduction of quality of life [37]. Thus, this study combined TyG index, neck circumference and ESS score. Although the predictive value is moderate (AUC 0.746,95% CI 0.701–0.701), it can be considered as a simple tool for rapid screening in outpatient clinics.

This study has some limitations. First, the nature of this retrospective study might compromise the conclusion. Second, the diagnosis of NAFLD in the study was made via the results of abdominal ultrasonography. When liver fat infiltration is < 20% or obese individuals, especially when BMI > 40 kg/m2, it is not reliable to detect steatosis [38–40]. However, compared to invasive liver biopsy, abdominal ultrasonography remains the preferred imaging method for NAFLD in clinical practice, as it is non-invasive and affordable [41].In the future, the prospective study can be carried out for snorer in different countries and regions to further enrich our conclusions.

In summary, the TyG index can highly indicate the risk of NAFLD in snoring patients. Meanwhile, a combination of TyG, neck circumferences and ESS score can serve as a convenient and effective predictive tool to screen NAFLD risk of snoring patients in the outpatient department.

## Data Availability

All data generated or analyzed during this study are included in this published article.
