# Publisher Correction: Triglyceride-glucose index and combined indicators: effective indicators for screening NAFLD in snoring patients

**DOI:** 10.1186/s12890-024-03209-0

**Published:** 2024-08-12

**Authors:** Yuqing Cai, Jia Chen, Xiaoyu Deng, Biying Wang, Jiefeng Huang, Ningfang Lian

**Affiliations:** 1 Department of Respiratory and Critical Care Medicine, Respiratory Disease Research Institute, The First Affiliated Hospital, Fujian Medical University, Fuzhou, 350005 China; 2 Department of Respiratory and Critical Care Medicine, National Regional Medical Center, Binhai Campus of the First Affiliated Hospital, Fujian Medical University, Fuzhou, 350212 China

**Publisher Correction: BMC Pulmonary Medicine (2024) 24:359**

10.1186/s12890-024-03166-8

Following publication of the original article, it came to the attention of the journal that the last author, Ningfang Lian, had been duplicated in the author list. The author list has since been corrected. The publisher thanks you for reading this erratum and apologizes for any inconvenience caused.

